# Contextualizing Public Health Interventions in Eliminating Endemic Diseases: New Lessons From a Review of Sri Lanka’s Success in Eliminating Malaria

**DOI:** 10.1177/00469580241308443

**Published:** 2024-12-18

**Authors:** Benjamin H. Wisden, Madhavi Hewadikaram, Uthsara Dissanayake, Curtis Maxwell, Poorna Gunasekera, Samuel Danso, Nuwanthi P. Katuwavila

**Affiliations:** 1 University of Plymouth, Plymouth, UK; 2 National School of Business Management, Colombo, Sri Lanka; 3 University of Sunderland, Sunderland, UK

**Keywords:** endemic diseases, epidemic, global health, historic, malaria control, malaria elimination, malaria eradication, new lessons, public health, review, Sri Lanka, timeline

## Abstract

Malaria remains a major global public health issue, demanding significant resources from governments, health organizations, and international organizations toward its elimination as an endemic disease. In 2016, Sri Lanka achieved the remarkable feat of being declared “malaria free” by the World Health Organisation (WHO), after over a century of indigenous disease. To identify significant lessons of global importance in eliminating endemic malaria by reviewing literature on Sri Lanka’s successful elimination campaign. The history of malaria in Sri Lanka highlights the nation’s journey from widespread malaria prevalence to achieving malaria-free status in 2016. Key interventions, such as the establishment of the Anti-Malaria Campaign in 1911, the introduction of Dichloro-Diphenyl-Trichloroethane (DDT) in 1946, and the launch of a malaria eradication program in 1958, played crucial roles in controlling the disease. However, challenges such as insecticide resistance, environmental changes, and civil war periodically caused resurgences. The 21st century saw intensified efforts in surveillance, vector control, and community engagement, culminating in the elimination of indigenous malaria cases in 2012. Despite this success, the risk of reintroduction from imported cases remains, necessitating ongoing vigilance and preventive measures. The case study of Sri Lanka is remarkable, and can provide valuable insight for stakeholders involved in eradicating malaria, with the caveat that this case is further evidence of the differential nature of malaria transmission worldwide.

**What do we already know about this topic?**
Malaria is a life-threatening global public health issue, the management of which has had varied success around the world. Sri Lanka, after over a century of differential patterns of incidence, was declared malaria free by the WHO in 2016.
**How does your research contribute to the field?**
This paper outlines the successes, failures, and challenges associated with malaria management strategies employed in Sri Lanka throughout the 20th century, up to today. Threats to Sri Lanka’s malaria free status are also discussed, as well as Sri Lanka-specific factors that may have contributed to the country’s successful elimination of indigenous malaria. The findings highlight the importance of contextualizing public health strategies in response to local challenges and resources to achieve sustainable success in eliminating endemic diseases.
**What are your research’s implications toward theory practice or policy?**
By providing a clear and accessible timeline of events occurring throughout Sri Lanka’s century long battle with malaria, this paper aims to provide stakeholders with a cohesive case study of successful malaria elimination, the lessons from which may be applied to other malaria endemic countries worldwide, with the eventual goal of worldwide malaria eradication. The emphasis on the contextualization of interventions in response to local challenges and needs paves the way for systematic incorporation of the Sustainable Development Goals into their design.

## Introduction

Malaria is a life-threatening disease caused by anthropophilic protozoan parasites of the genus *Plasmodium*. It is primarily caused by *Plasmodium falciparum* in Africa, and P. *vivax* in countries outside the African continent.^1^ It is transmitted to humans through bites of female *Anopheles* mosquitoes. Despite being treatable and curable, malaria remains a considerable public health concern worldwide, and notable efforts have been made globally toward elimination. Malaria is endemic to 85 countries and territories. Whilst the highest rates of transmission are found in sub-Saharan Africa, transmission rates are also moderately high in areas of central and South America, Asia, and parts of Oceania. According to the WHO’S 2023 world malaria report,^2^ the global prevalence of malaria was 249 million cases, an increase of 5 million new cases over the previous year. Based on WHO estimates, rates of new malaria cases have increased globally since 2015, with the largest increase (an additional 11 million cases) occurring between 2019 and 2020. Additionally, estimated deaths have increased by approximately 22 000 since 2015.

In 2016, Sri Lanka became one of the first countries in South Asia to be declared malaria free by the WHO.^3^ This essay aims to discuss the history of malaria control in Sri Lanka, with a view to providing stakeholders with an excellent example of a country that was able to eliminate malaria despite significant biological, logistical and environmental challenges.

## Methodology

A literature search was conducted using Google Scholar as a primary database. This database was selected due to its capability to sort by relevance and prioritize frequently cited articles. The search included case studies, reviews, and historical analyses, encompassing published articles on trends in the global, regional, and national incidence of malaria and the management and eradication of the disease by control measures. Search terms primarily included “malaria in (relevant year) in Sri Lanka,” where historical articles, reports and case studies were selected, along with WHO and Sri Lanka’s Anti-Malarial Campaign (AMC) reports. Given the historical nature of this essay, search criteria encompassed articles and reports published between 1905 and 2024. This is due to malaria first being reported as endemic in Sri Lanka in 1906. Published articles relating to the broader subject of malaria in Sri Lanka are numerous—including around 49 000 initial hits, and due to the non-systematic nature of this review, sources were selected based on relevance to Sri Lanka, credibility, and number of citations. Selected sources relate to specific events in the history of malaria in Sri Lanka, for example the introduction of the insecticide “malathion,” and outcomes of reports published by the AMC and WHO. Each key historical event was searched, and the most useful articles selected based on the scrutiny of the authors. Within articles, relevant citations were investigated to assess credibility and usefulness, and included or excluded thusly.

## Body

### A Brief History of Malaria in Sri Lanka

Sri Lanka is an island located in the Indian Ocean, south of India, and has a population of around 23 million.^4^ The island is divided by a central hilly region into “wet” and “dry” zones, based on annual rainfall.^5^

Earliest known records of malaria-like disease in Sri Lanka come from 13th century Buddhist texts, and may have coincided with the building of early dams and canal systems, intended to bring water to the dry regions.^5^ Later documents referring to malaria-like disease were produced during the Dutch occupation of Sri Lanka between the 17th and 19th centuries, with maps reporting “febrile illnesses” within both dry and wet zones.^5^ By the early 1900s, malaria was well established in Sri Lanka, then known as Ceylon, with a spate of epidemics occurring throughout the entirety of the 20th century, including 1906, 1914, 1919, 1923, 1934, 1967, and 1986.^6^ A timeline of key events in the history of malaria in Sri Lanka will now be detailed, in order to outline how this nation was able to progress from a highly deprived, malaria endemic island, to being declared malaria free in just over 100 years.

### 1911: First Anti Malaria Centre Set Up

The establishment of the Anti-Malaria Campaign (AMC) in 1911, and the construction of the nation’s first anti-malaria center could be considered to represent the first state-organized intervention into malaria control of the modern era.^7^ Measures to control malaria at the time were limited to basic environmental manipulation for vector control such as filling, drainage, and “oiling” of standing water used for breeding of *Anopheles culcifacies*, and the first malariologist was not appointed until 1921.^7^

### 1921: Appointment of First Specialist Malariologist

In 1921, the incidence of malaria in Sri Lanka was around half a million cases.^8^ Henry F. Carter became the first state appointed malariologist in the country. Numerous control efforts were put into place on his advisement. Firstly, larvivorous fish were introduced to confirmed and potential breeding sites for *Anopheles*, and vector control efforts were refocussed, with particular emphasis being placed on areas under the remit of the Ceylon Estates Proprietary Planters’ association.^7^ Additionally, widespread malaria education was provided to the Sri Lankan populace during this time.^8^ Another key measure in malaria control during this period was chemical larviciding,^8^ with chemicals used including Shell Malariol and Paris Green.^8^ Regular chemoprophylaxis with quinines, medication highly toxic to *Plasmodium*, was also introduced during this period.^9^ Government investment in malaria control was also stepped up during this decade,^10^ including increased funding toward sanitation and water supply. Despite these measures, the largest malaria epidemic in Sri Lanka’s history was yet to come.^8^

### 1934-35: A Devastating Epidemic

A prolonged period of unusually low rainfall throughout 1934 resulted in formation of pools of standing water, expanding available habitats for *Plasmodium* vectors, principally *A. culcifacies*.^11^ This resulted in expansion of the range and intensity of this epidemic, totaling almost 80 000 deaths in an infected population of around 5 million.^5,11^ There is evidence to suggest that the over-bureaucratization of the healthcare system in Sri Lanka was also a factor in the delayed initial response to the crisis.^12^ The department of healthcare was under intense cost-cutting scrutiny, and medical personnel were reluctant to act without official sanction.^12^ Eventually, colonial Britain scaled up provision of aid, with quinine, food, and funding being provided. By 1937, malaria incidence had decreased to around 2 million cases.^11^ In the years following this epidemic, an organized system of entomological surveillance was set up nationwide, with professional mosquito catchers being employed on a monthly basis to provide reports on the breeding habits and movement of potential vectors.^13^ In spite of this, malaria incidence in Sri Lanka would continue to oscillate between around 1.8 million and 3.5 million cases for approximately the next decade, until the next major intervention in 1946.

### 1946: DDT is Introduced

After a limited trial of spraying of the insecticide dichloro-diphenyl-trichloroethane (DDT) in the Kekirawa region in November 1945, DDT spraying was expanded in phases, and was nationwide by 1947.^14^ The vast majority of the dry zone, the entire intermediate zone, and malaria endemic areas in the wet zone were treated, with houses being sprayed periodically with DDT to coat surfaces that mosquitoes were likely to rest upon. This indoor residual spraying (IRS) proved extremely effective, with cessation of malaria transmission seen in both wet and intermediate zones,^7^ and was followed by progressive interruption of spraying in following years,^15^ partially due to reports of DDT resistance being developed in mosquitoes in other countries.^7^ By 1958, the number of malaria cases had dropped to 1037, from over 10 000 the previous year.^7^

### 1958: Malaria Eradication Programme is Launched

After the massive success of the DDT IRS scheme for reducing malaria incidence in Sri Lanka, and the resolution of the eighth World Health Assembly, the Sri Lankan government accepted a proposal for a malaria eradication program. Headquartered in Colombo, this “Anti Malaria Campaign” aimed to remove the parasite reservoir for malaria in Sri Lanka within 5 years.^7,16^ The dry zone was placed immediately into an “attack phase”^16^ where IRS was resumed, and intermediate and wet zones placed in a “consolidation phase” due to cessation of transmission in those areas, with IRS not being required. Entomological surveillance was stepped up, and reporting of malaria cases codified into a legal obligation.^16^ From this point forward, effective malaria surveillance will form a cornerstone of efforts that lead to the eventual elimination of the disease within Sri Lanka. Between 1958 and 1963, annual blood examination rate (ABER) and annual parasite incidence (API) were both within the recommended ranges set by the WHO, with 1963 representing a record low of only 17 cases.^8,16^

### 1967-1968: A New Epidemic

After cessation of IRS in 1964, foci of *P. falciparum and P. malariae* infection reappeared, increasing in number over the subsequent 3 years. Between July and September 1967, 800 cases of malaria infection were reported, with the principal parasite being *Plasmodium vivax*, an organism that had had negligible impact in previous malaria epidemics.^17^ This is believed to represent a previously unknown reservoir of infection in the population, with cases being detected mainly in new development projects and farming settlements in dry and intermediate zones.^17^ Centers of population aggregation such as the Elehara gem mining center and Kataragama religious center were invaded by cases of *P. vivax* infection, which then was spread to other population centers, eventually being disseminated throughout the entirety of the malarious regions of Sri Lanka.^17^ Estimates of total incidence of malaria during this epidemic range from around 500 000 to around 1 500 000, with around three-fifths of the nation being affected, in all 3 zones.^11,17^ In spite of this resurgence of infection, only 58 deaths related to malaria were reported.^17^ This is thought to be due to infections being caused overwhelmingly by *P. vivax* during this period, a pathogen with significantly less virulence than other *Plasmodium* species.^18^ In spite of the significantly reduced mortality and morbidity associated with this epidemic when compared to the devastating epidemic of 1934/35, the resurgence of malaria after a period of near elimination represented a warning sign to the Sri Lankan government that their handling of the gradual reduction in cases through the 1960s, decreased vigilance, reduction in IRS and disbandment of spraying teams, and decreasing vector control measures, was premature. In ensuing years, numerous small peaks of incidence would continue to occur.

### 1969-1980: Insecticide Resistant A. culcifacies and Quinine Resistant P. falciparum Develop

Prior to 1969, DDT remained the most effective insecticide against Anopheline mosquitoes, confirmed by susceptibility reports published by the Anti Malaria Campaign in 1967, Anti Malaria Campaign in 1968.^19,20^ However, by the start of 1969, 4 centers reported that varying degrees of resistance to DDT had been detected within *A. culcifacies* specimens.^7^ This was confirmed officially in susceptibility reports published the same year.^21^ During the period from 1974 to 1975, malaria incidence increased rapidly in several areas of Sri Lanka, with *P. falciparum* incidence also markedly increasing.^7^ This increase in incidence has been ascribed to the spread of DDT resistant *A. culcifacies* throughout the country, prompting action from the Anti Malaria Campaign. Malathion, an organophosphate insecticide unrelated to DDT, and therefore unaffected by the recently developed resistance among *A. culcifacies* vectors, was implemented in areas of high incidence, before being rolled out to the wider country, replacing DDT entirely in the IRS program in 1977.^7,11^ Whilst this measure was initially promising, malaria incidence continued to rise through the 1980s.^6^ Between 1982 and 1984, 275 000 cases were reported, affecting both dry and intermediate zones. Whilst initial explanations posited the idea that malathion resistance was being developed in *A. culcifacies*, poor quality IRS was found to be of much more significance, and this epidemic was eventually explained as being due to a combination of the operational failure of IRS throughout the affected regions combined with poor rainfall, in an AMC report.^7^ In 1986, a significant rise in malaria incidence was recorded, and by 1987 almost 700 000 cases had been positively reported. Of these cases, a significantly higher proportion were caused by *P. falciparum* than previous years, with *falciparum* malaria representing 27% of cases, compared to only 2.5% 3 years prior.^22^ This was attributed to the increasing spread of chloroquine resistant *P. falciparum*, which had first been detected in 1984.^23^ New irrigation developments also brought non-immune populations into malaria endemic zones, increasing transmission.^7^

### 1990s: Landmark Malaria Control Developments Against a Backdrop of Civil War

Through the early 1980s, ethnic rioting and other low-level insurgency prompted violent reprisals, leading to the development of a separatist movement in Sri Lanka that threw the nation into civil war. Violent conflict would continue to exist for the next 26 years, causing significant displacement of Sri Lankan populaces, disruption of infrastructure responsible for delivery of medicine and significant mortality.^24^ Populations living in conflict zones controlled by separatist groups represented significant reservoirs of malaria, with rates of infection being proportionally higher than the rest of the country.^25^ Both separatist groups and the Sri Lankan government therefore had motivation to prevent a resurgence of malaria, and unofficial, followed by official ceasefires were organized throughout the 1990s in order to provide opportunities for immunizations and unimpeded provision of medical supplies and infrastructure.^25^ Regional malaria officers (RMOs) in areas neighboring conflict zones coordinated provision of medical supplies, along with stakeholders such as Red Cross and Sarvodeya.^25^ A longer period of ceasefire between 2002 and 2006 enabled more efficient health infrastructure, which aided the AMC effort.^13^

Changes to the structure of the AMC occurred during this period. In 2001, Many responsibilities of the previously centralized AMC were relinquished to provincial Ministries of Health,^5^ allowing for provision of malaria control measures in a way that was better tailored to each area’s individual need during the conflict.

Malathion resistance, first detected in malaria vectors in 1982,^26^ led to its discontinuation for indoor residual spraying (IRS) in 1993.^27^ Malathion was replaced primarily by the synthetic pyrethroid λ-cyhalothrin, although cyfluthrin, deltamethrin, etofenprox, and fenitrothion were also used. The rollout of these insecticides, which were a combination of pyrethroids and organophosphates, was done on a rotational basis from one district to the next, with the intention being to delay the development of resistance to these new insecticides among *Anopheles* species.^27^ An additional positive effect of this change was increased uptake of IRS by the general populace, most likely due to the fact that the new insecticides were odorless and did not leave residue on the surfaces on which they were sprayed.^13^ A significant shift in the practice of IRS also occurred, with the original strategy of “global” IRS being switched to the practice of targeted IRS between 1996 and 1997.^13^ Areas of historical transmission, areas with increased proximity to vector breeding sites, areas of increased incidence of *P. falciparum*, and areas with confirmed chloroquine-resistant infections were targeted.^13^ Long lasting insecticide treated nets (LLITNs) treated with pyrethroids were also distributed throughout the country by the AMC during this period.

Parasitological surveillance was also increased during the 1990s, with active case detection (ACD) being introduced in 1997 with funding from the World Bank.^13^ This practice, defined as “detection by health workers of malaria infections at community and household level in population groups that are considered to be at high risk” by the WHO,^28^ involves screening for fevers in groups with high malaria risk. All febrile populations, or at-risk populations without prior screening, are then given full parasitological examination including blood film microscopy, in order to detect asymptomatic or symptomatic carriers of *Plasmodium*.^29^

In spite of these myriad developments, malaria incidence continued to rise throughout the 1990s, from 142 294 cases in 1995 to 265 549 in 1999.^30^ It should be noted, however, that only 102 deaths eventuated from said cases in 1999.^31^

### Into the 21st Century

From 2000 to 2001, malaria cases reduced by 68%, followed by a 38% decrease from 2001 to 2002. A 75% decrease was noted the subsequent year.^32^ According to a study published in 2019, this was due to “Intensified parasitological surveillance focusing on early diagnosis and treatment, entomological surveillance, selective vector control, enhanced health awareness, and community engagement programmes [which] were carried out by the Anti Malaria Campaign.”^33^ In 2003, funding was granted to the AMC for malaria control by the Global Fund to fight AIDS, TB, and Malaria (GFTATM), equating to over 7 million U.S. dollars. Further grants would be disbursed in 2005, 2009, and 2016.^34^ Malaria deaths also decreased during this period, with 88 deaths due to indigenous malaria being reported between 2001 and 2004.^31^ By 2004, malaria incidence in Sri Lanka was at “pre-elimination” levels according to the AMC, with the caseload representing fewer than 1 infection per 1000 members of the population. In spite of this, the AMC opted to delay moving into an “elimination” phase, due to logistical issues continuing to be presented by the civil war.^33^ By 2008, the proportion of malaria caused by *P. falciparum* had reduced to only 8% of cases, from 40% of cases in 2000,^5^ and the same year the AMC formally moved into the pre-elimination phase, with an objective of interrupting *P. falciparum* and *P. vivax* transmission by 2013 and 2015 respectively. Individual case reporting was also introduced to the AMC in 2008, with 24-h reporting of malaria cases conducted by RMOs, and information stored in an electronic malaria elimination surveillance database. This allowed for 24-h analysis of trends in malaria transmission, aiding elimination efforts significantly, with the intention being to eventually merge this data with the national database for other diseases.^13^

In 2009, the civil war in Sri Lanka ended, significantly improving the ability of the AMC to deliver interventions nationwide.^33^ Due to this, and the efforts previously mentioned, the AMC moved officially into the elimination phase in 2011, a year which recorded only 124 cases nationwide.^33^

After a century of continuous effort by the AMC, Sri Lanka reported its last indigenous malaria case in 2012.^35^

### A Malaria Free Sri Lanka

After 3 consecutive years with no indigenous cases, Sri Lanka was declared malaria free by WHO in September 2016.^11^ However, eager to avoid repeating the mistakes of the past in scaling down measures too early, rigorous case detection, entomological and parasite surveillance continued to be employed by the AMC.^5^ Cases of imported malaria continued to be detected from returning overseas travelers. 95 cases of imported malaria were detected in 2013, and 49 in 2014. AMC investigation showed that these imported cases were represented by 2 specific clusters of infection, namely Pakistani asylum-seekers who moved into a malaria non-endemic area of Sri Lanka, and local fishermen returning from Sierra Leone.^36^ Despite the enormous achievement of malaria elimination, clusters of imported disease such as these represent the current biggest threat to Sri Lanka’s malaria-free status, namely the threat of re-introduction.

### Prevention of Re-introduction of Malaria in Sri Lanka—The Current Situation

Between the last indigenous case of malaria in 2012 until the end of 2023, 532 imported cases have been reported, approximately 50 per year.^37^ There was also a case of introduced malaria in 2018, where a recently imported malaria case spread to a local,^38^ and a case of transfusion induced malaria in 2021.^39^ As such, early detection and management of imported malaria cases remains the highest priority for the AMC, who published guidance in 2023 on this point.^40^ Among the points included in the guidance, rigorous investigation of suspected parasitemia in travelers returning from malaria endemic countries, and a low threshold of clinical suspicion in patients presenting with malaria symptoms were cornerstones.^40^

Voluntary malaria screening centers are operational in ports of entry to Sri Lanka, and collaboration between the AMC and the Sri Lankan Army has made screening of returning UN security personnel simple. Surveillance within other groups such as Pakistani asylum-seekers, overseas workers on tourist visas, and immigrants who go undocumented as residents has presented a greater challenge.^36^ The logistical cost of presumptively treating all “high risk” migrants, potential lack of uptake, and risk of creating an ethnic “out-group” due to labeling certain groups as “at risk,” make this an option of questionable merit.^36^

Sri Lanka’s “whole of government” framework entails seamless integration of various government bodies, with army, air force, navy, and police personnel all playing a part in protecting Sri Lanka’s malaria free status. The AMC works closely with these bodies, as well as different government ministries, international organizations, private stakeholders, and private institutions such as dockyards and fisheries who may have a significant number of foreign workers and can contribute to blood screening.^41^ Community engagement has also played a big role, with the AMC establishing a public health service network via which public engagement is conducted. This includes malaria education in schools, and engagement with news outlets such as radio, television, and newspapers, to deliver up to date malaria information to the public. This education focusses on general “public awareness” of malaria signs and symptoms, as well as “personal awareness,” focusing on helping the public reflect on the personal ramifications of visiting malaria endemic countries.^41^ Through these measures, the public’s role in engaging with the fight against malaria has grown.

### Current and Future Threats to Sri Lanka’s Malaria-Free Status

In 2023, the Asia Pacific Leaders Malaria Alliance (APLMA) published a report on the state of malaria control in Asia,^41^ outlining current threats to the malaria free status of Sri Lanka. Some of these will now be briefly discussed.

**Disruption in healthcare infrastructure:** Anything that disrupts the provision of healthcare, delivery of supplies, access to key institutions for surveillance etc. could wreak havoc on the national malaria program. This could include natural disasters, as seen in the 2004 earthquake, conflict such as civil war, or political instability. Issues such as these can often be overcome, as seen during the civil war and the COVID pandemic of 2019-2023,^42^ but the current malaria-free status of Sri Lanka is dependent on a health service that is cohesive, with capacity for on the button surveillance and rapid diagnosis and follow-up.

### Scaling Back of Surveillance and Control Efforts, Reduction in Vigilance

As seen in the malaria epidemic of 1967, low malaria incidence should not be taken for granted, and scaling back of any malaria control measures should not occur without scrutiny and sufficient infrastructure to rapidly resume surveillance if need be. Without efficient monitoring of potentially malarious areas and prompt management of any newly detected cases, resurgence may occur. Continuing use of appropriate insecticides, larval source management, and reporting of potential vector populations must continue.

**Changes to political commitment and/or funding:** If the malaria program loses political commitment, especially relating to a perceived notion that the problem of malaria has been “solved,” or funding dries up, malaria control may be compromised. Continuing lobbying, education about the risks of malaria resurgence, and continuing international support may help to combat this risk.

**Insecticide/drug resistance:** As seen during the mid to late 20th century, development of resistance to insecticides and antimalarials is a very real danger and can lead to significant rises in malaria incidence. In the context of Sri Lanka today, this represents a risk of resurgence of malaria in previously well controlled areas. Maintaining rotational insecticide usage and not over-extending use of antimalarial medication will help combat this, in combination with vigilant susceptibility monitoring.

**Vector related changes:** New reservoirs of vector breeding, or changes in the ability of vectors to transmit specific species of *Plasmodium* may lead to a resurgence of malaria. Given that malaria relies on competent mosquito vectors for transmission, continuous entomological surveillance and control is emphasized.

**Loss of acquired immunity:** Acquired immunity to malaria may start to wane, now that indigenous malaria has been eliminated in Sri Lanka. As such, previously immune populations may start to show more susceptibility to malaria, increasing the importance of prompt diagnosis and intervention within at-risk groups.

**Changes in the** “**human factor**”: There are myriad “human” factors involved in the control, or resurgence, of malaria. Firstly, public perception of the problems caused by malaria may start to decrease in line with the reduced impact malaria has had on the Sri Lankan populace since elimination. This complacency may reduce the populace’s malaria vigilance, engagement with healthcare, and compliance with preventative measures, potentially leading to resurgence.

**Environmental changes:** Certain human-influenced environmental changes brought on by deforestation, irrigation schemes, urbanization etc. can be drafted with malaria as a consideration. As seen in the case of the Mahaweli irrigation project, manmade ecological changes can create hotspots of malaria transmission, both due to influx of malaria naïve populations, and creation of vector reservoirs. Future developments such as these should therefore be assessed in line with current malaria control recommendations, and adequate measures taken to reduce the risk of resurgence. Certain ecological changes such as reduction in rainfall may also increase risk of malaria transmission, and care should be taken to prepare for these eventualities.

## Conclusions—What Can the World Learn About Malaria Elimination From Sri Lanka?

As this essay has outlined, myriad factors combined to create the environmental, biological, political, and social context in which Sri Lanka has been able to achieve the staggering feat of malaria elimination. The lessons learned from the major epidemics of 1934 and 1967 have provided valuable insight into the effects of environmental changes on vector borne disease, as well as the importance of maintaining control efforts when disease incidence is loBw. This is doubly pertinent in the current climate, with over 100 countries having eliminated malaria in the last century, 25 having done so since 2000.^43^ The evolution of the AMC from a single colonial era malaria center to the decentralized, highly integrated force it is today, with its network of parasitological and entomological surveillance, diagnosis and treatment centers, public education, and many more facets, forms a highly useful case study of how government sanctioned public health initiatives develop. The AMC’s efforts in controlling malaria against a backdrop of decades long conflict also provides a case study of how to efficiently manage this significant challenge, which prove useful given the prevalence of infectious disease in current conflict zones such as Gaza,^44^ Ukraine,^45^ Lebanon,^46^ and many more. Other malaria-endemic regions such as India and Sub-Saharan Africa have employed broadly similar strategies for controlling malaria. LLITNs and IRS have been employed in both regions over the last 25 years,^47-49^ and yet malaria incidence and mortality remains high. In Sub-Saharan Africa, although IRS and ITN use have increased considerably in recent years, there is limited evidence of rotation between insecticide classes, a strategy that was widely employed in Sri Lanka. This represents one example of how operational variability remains within malaria control strategies between regions, and how local costs and organizational challenges can influence the success of said strategies.

Following this, some factors specific to Sri Lanka that may have played a role in the success of the AMC in eliminating malaria when compared to other countries should be mentioned. Firstly, Sri Lanka is an isolated island, with the routes of ingress being mostly limited to official ports of entry, an environment in which malaria surveillance records are relatively easier to maintain than other countries with long and potentially unmonitored land borders. Secondly, Although Sri Lanka showed significant rates of transmission in certain “hotspots,” rates of transmission in other areas of the country were significantly lower than other malaria endemic countries, such as those in sub-Saharan Africa.^5^ This may be due to the prevalence of different vector species, with different ecological requirements for transmission, and differences in *Plasmodium* species proportion. On this point, other parts of the world show other differences in availability of vectors for *Plasmodium.* For example, malaria transmission via monkeys has been observed in South America.^50^ These factors, in combination with others such as Sri Lanka’s relatively small population, relative ease of public access to medical facilities in recent years, relatively high proportion of government malaria spending, and high level of freely available education, make Sri Lanka a nation that, in spite of historic challenges, was well equipped to combat the challenges implicit in the task of malaria elimination. The case study of Sri Lanka is remarkable and can provide valuable insight for malaria stakeholders globally, with the caveat that this case is further evidence of the differential nature of malaria transmission worldwide. As the AMC did with Sri Lanka, organizations in other endemic countries must recognize the specific challenges associated with their nation’s ecology, population, political situation, resistance levels etc. By doing so, we can work toward the vision of a malaria free world.

## Was There a Hidden Magic Bullet?

Despite all the peculiarities highlighted above, the overall progress of malaria within the island broadly mirrored what has manifested in other endemic countries with alternating periods of remission and epidemics, driven by a multitude of factors such as emergence of resistance, changes of habitat, migration, and commitment of state/human personnel. Is there any singular point within the timeline of malaria in Sri Lanka which could point to a hitherto unrecognized “magic bullet” that resulted in a major and seemingly irreversible change in its trajectory? What seems remarkable in the timeline is the rapid reductions recorded in the incidence and prevalence of the disease at a time when the island was plagued with civil strife which meant significant parts of the country were not under direct state control. Yet, it is within that self-same period that the AMC underwent significant restructuring in 2001, with many central responsibilities being transferred to regional bodies.

This meant regional authorities could not only focus their efforts to local priorities, but they were also able to allocate vital resources based on local needs. This in turn may also have led to more community engagement, which has been highlighted as a strength in this success story. “Contextualizing” malaria control by providing populations with the capacity to respond to local threats, paved the way for shared ownership of elimination efforts with those who were most exposed to the risks of the disease.

Even as we as a global community are seeking universal remedies for equity through the Sustainable Development Goals, could the Sri Lankan success story point to the importance of systematic decentralization through the upholding their founding principles of (1) leaving no one behind and (2) reaching the furthest behind first, as key fundamentals?
